# Elastin in the Liver

**DOI:** 10.3389/fphys.2016.00491

**Published:** 2016-10-25

**Authors:** Jiří Kanta

**Affiliations:** Department of Medical Biochemistry, Faculty of Medicine in Hradec Kralove, Charles University in PragueHradec Kralove, Czechia

**Keywords:** elastic fibers, microfibrils, elastin, fibulin, hepatic stellate cells, portal fibroblasts, TGF-β, liver fibrosis

## Abstract

A characteristic feature of liver cirrhosis is the accumulation of large amounts of connective tissue with the prevailing content of type I collagen. Elastin is a minor connective tissue component in normal liver but it is actively synthesized by hepatic stellate cells and portal fibroblasts in diseased liver. The accumulation of elastic fibers in later stages of liver fibrosis may contribute to the decreasing reversibility of the disease with advancing time. Elastin is formed by polymerization of tropoelastin monomers. It is an amorphous protein highly resistant to the action of proteases that forms the core of elastic fibers. Microfibrils surrounding the core are composed of fibrillins that bind a number of proteins involved in fiber formation. They include microfibril-associated glycoproteins (MAGPs), microfibrillar-associated proteins (MFAPs) and fibulins. Lysyl oxidase (LOX) and lysyl oxidase-like proteins (LOXLs) are responsible for tropoelastin cross-linking and polymerization. TGF-β complexes attached to microfibrils release this cytokine and influence the behavior of the cells in the neighborhood. The role of TGF-β as the main profibrotic cytokine in the liver is well-known and the release of the cytokines of TGF-β superfamily from their storage in elastic fibers may affect the course of fibrosis. Elastic fibers are often studied in the tissues where they provide elasticity and resilience but their role is no longer viewed as purely mechanical. Tropoelastin, elastin polymer and elastin peptides resulting from partial elastin degradation influence fibroblastic and inflammatory cells as well as angiogenesis. A similar role may be performed by elastin in the liver. This article reviews the results of the research of liver elastic fibers on the background of the present knowledge of elastin biochemistry and physiology. The regulation of liver elastin synthesis and degradation may be important for the outcome of liver fibrosis.

## Introduction

Elastin is the most stable protein of the extracellular matrix (ECM) which is composed of collagen, glycoproteins, glycosaminoglycans and proteoglycans (Aumailley and Gayraud, 1998). It is present in large amounts in the organs whose elastic properties are essential for their function such as arteries and lungs. Elastin is synthesized around the birth when these organs begin to function and its synthesis subsides in the postnatal period. Most of the protein persists in the body for the lifetime (Shapiro et al., 1991). It may be degraded and resynthesized in various organs in pathological conditions. Elastic fibers appear in the scar in later stages of wound healing (Raghunath et al., 1996; Sproul and Argraves, 2013). Elastin accumulates in fibrotic livers regardless of fibrosis etiology (Liban and Ungar, 1959) and because of its low turnover it may contribute substantially to the irreversibility of the disease.

Elastic fibers are composed of the insoluble elastin core, microfibrils on the surface of the fibers and a number of attached proteins that help to assemble the fiber (Kielty et al., 2002). Elastic fibers are not an inert component of the ECM. Elastin precursor tropoelastin, elastin polymer and elastin degradation products all possess biological activity that may influence inflammatory cells and connective tissue cells in the organ (Almine et al., 2012). Moreover, the microfibrils bind the cytokines of the TGF-β superfamily that may influence the course of liver fibrosis, regeneration and cancer (Bissell et al., 2001; Dooley and ten Dijke, 2012).

The present review summarizes the results of elastin research in the liver on the background of the present knowledge of elastin biochemistry and physiology. The aim of this review is to encourage further studies of elastin and other components of elastic fibers in the liver because it may help to solve the problem of cirrhosis irreversibility.

## Elastin core of elastic fibers

**Figure 1:**
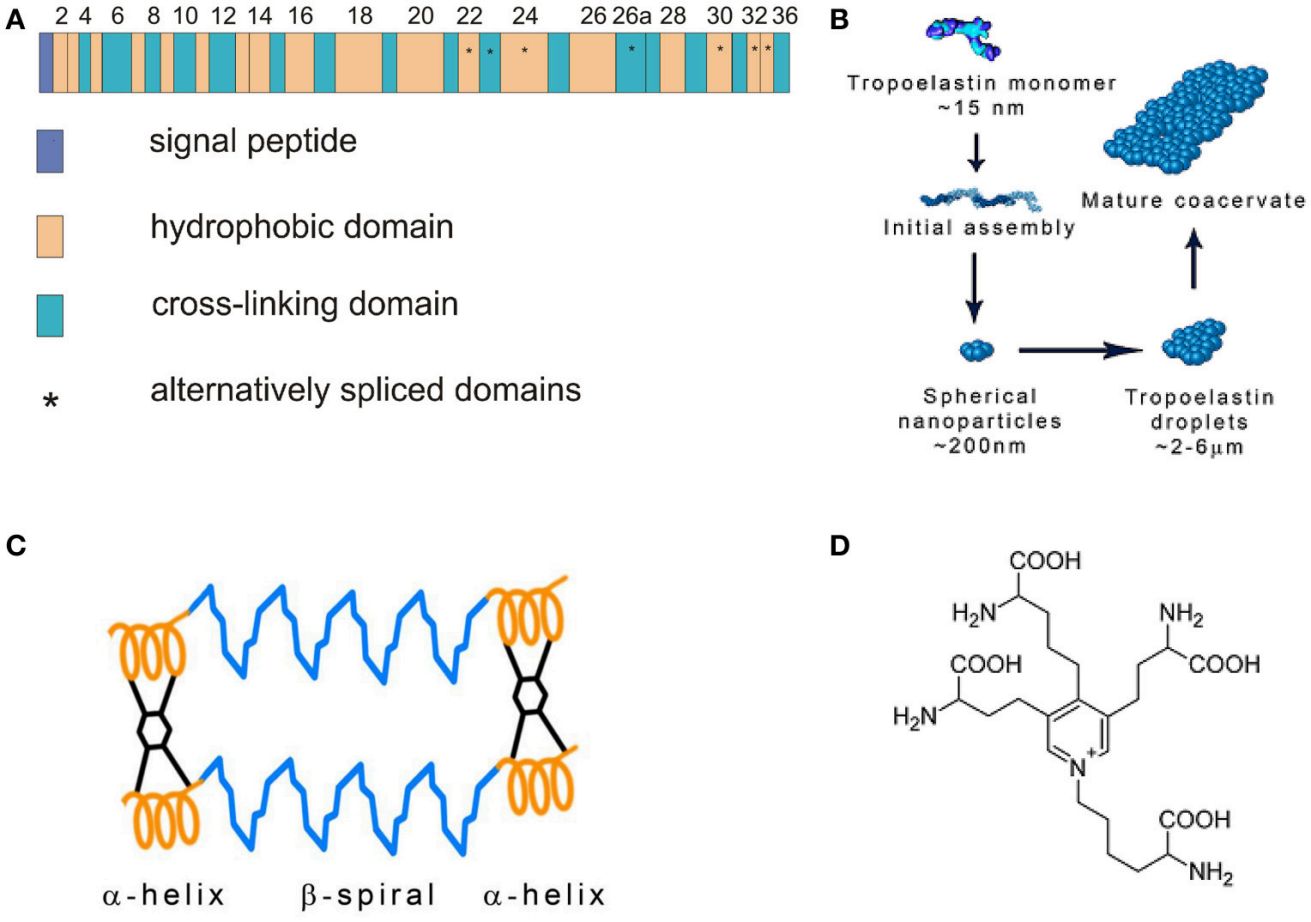
**(A)** Tropoelastin gene structure. Modified from Mithieux and Weiss (2005). **(B)** Tropoelastin (TE) assembly. TE molecules polymerize in a head-to-tail mode and subsequently coacervate. Modified from Wise et al. (2014). **(C)** Fibrillar model of elastin. Tropoelastin chains are connected with desmosine cross-links. Modified from Vrhovski and Weiss (1998). **(D)** Desmosine molecule.

Elastic fibers are composed of the elastin core and the microfibrillar mantle upon which is elastin deposited. The soluble monomer of elastin is tropoelastin (TE) possessing a molecular weight of about 72 kDa. TE is a single gene protein encoded by 36 exons that persist in many animal species. Exons 34 and 35 were lost during the evolution of human TE. Seven other exons may be alternatively spliced reflecting elastin function in different tissues (Mithieux and Weiss, 2005; Figure 1A). The exons vary in length from 27 to 114 base pairs and code for domains with alternating hydrophobic and hydrophilic structures. Hydrophobic regions contain nonpolar amino acids glycine, alanine, valine, proline, leucine and isoleucine, are arranged in repeating motifs such as GVGVAP and tend to form β turns. Hydrophobic regions confer elasticity to the protein. Hydrophilic domains are rich in lysine and alanine and tend to form α helices; lysine residues are separated by two or three alanine residues, e.g., AAKAAAKAA. The lysine residues in the hydrophilic regions give rise to cross-links that connect neighboring TE molecules. The interaction of four lysine residues in a specific spacial arrangement is required for the synthesis of desmosine and isodesmosine, the crosslinks characteristic of elastin (Rosenbloom, 1984; Vrhovski and Weiss, 1998; Wise et al., 2014).

Human TE is composed of 760 amino acid residues. It contains the N-terminal signal sequence that is removed when the newly synthesized protein enters the endoplasmic reticulum. TE interacts with the elastin binding protein (EBP) which is an enzymatically inactive variant of β-galactosidase. EBP functions as a 67 kDa chaperone that prevents intracellular TE aggregation and protects TE from proteolytic degradation. TE is packaged into secretory vesicles in Golgi apparatus (Mithieux and Weiss, 2005). After secretion into the extracellular space, TE molecules rapidly associate in a head-to-tail mode and assemble on the cell surface. This process is called coacervation (Figure 1B). The cells interact transiently with elastic fibers and globular elastin is transferred to the fibrillin containing microfibrils on the surface of the fibers (Kozel et al., 2006). The coacervation is promoted by interactions of specific regions of TE and fibrillin-1, the component of microfibrils (Clarke et al., 2005). Sialidase (neuraminidase-1) bound to EBP removes the terminal sialic acid from the carbohydrate chains of microfibrillar glycoprotein and facilitates TE binding. EBP dissociates from TE and most of it is recycled (Hinek, 1996; Debelle and Tamburro, 1999; Hinek et al., 2006).

The stabilization of coacervates on the microfibrillar scaffold requires initial oxidation of specific lysine residues and TE crosslinking (Sato et al., 2007). Protein-lysine 6-oxidase (lysyl oxidase, LOX), a copper containing enzyme, oxidizes lysine residues to α-amino adipic δ-semialdehydes (allysines) that may spontaneously interact. They form an aldol in a condensation reaction or a Schiff base when an allysine reacts with an intact lysine residue. These compounds are intermediates in the synthesis of elastin cross-links lysinonorleucine, desmosine and isodesmosine that connect neighboring TE chains (Figures 1C,D). Lysine oxidation requires enzymatic catalysis, the subsequent reactions are spontaneous (Rucker et al., 1998). TE is easily degraded by proteinases but crosslinking makes it a highly stable polymer resistant to proteolysis (Maurice et al., 2013).

## Elastin in healthy and fibrotic liver

Elastic fibers are found in arteries, lungs, skin, elastic ligaments and bladder—the organs subjected to stretching and expansion. However, elastin networks can also be detected in tissues whose primary function is not to provide elasticity and resilience, e.g., articular cartilage and adipose tissue (Green et al., 2014).

The use of orcein staining in histochemistry made it possible to study elastic fibers in normal and cirrhotic liver. According to Hohenemser (1895) newly formed elastic tissue in cirrhotic liver is found around the main branches of the hepatic artery and the portal vein. The fibers are less numerous in the neighborhood of fine branches of these vessels and in the acini. They surround bile ducts. Hohenemser noticed two important features of liver fibrosis. First, connective tissue in cirrhotic liver resembles the connective tissue found in the scars formed in healing wounds in the skin and other organs and elastic fibers are a result of the scar development. Second, fibrotic septa are a product of new connective tissue synthesis (Hohenemser, 1895). Thin elastic fibers are found in the liver capsule and in the portal areas of normal liver. Elastic fibers are found in enlarged portal spaces and in the internodular fibrous septa in fibrotic liver. Patches of elastin are present in the walls of sublobular and central veins. Hyperplasia of elastic fibers is increased in fibrotic liver regardless of the origin of the disease. Staining with orcein, aldehyde fuchsin, orcinol-new fuchsin and resorcin-fuchsin gives similar results (Liban and Ungar, 1959). The results yielded by classical staining can be confirmed by immunohistochemistry and electron microscopy (Porto et al., 1990a).

**Figure 2:**
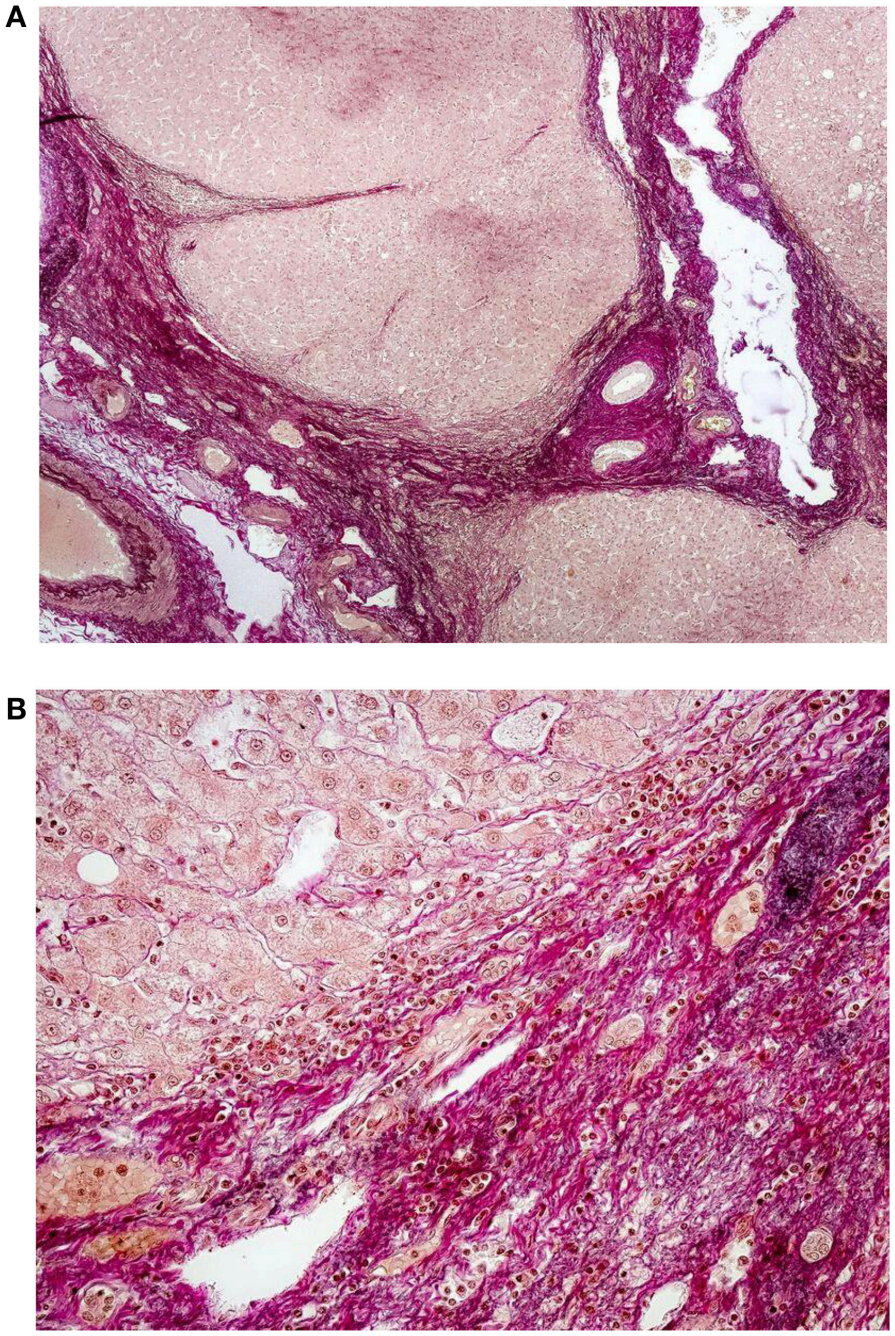
**(A)** Histology of liver cirrhosis—overview. Severe cirrhosis (magenta color) separates regenerative nodules of hepatocytes. Preexisting structures of bile ducts, portal vein branches and hepatic artery are entrapped in the fibrotic areas. Focal deposition of elastic fibers (black color) is visible; van Gieson elatic staining, 40x. (Ryška and Nová, Unpublished results). **(B)** Detail of marked periportal fibrosis in cirrhotic liver with deposition of fine elastic fibers. Some of the fibers irradiate into the regenerative nodules of hepatocytes; van Gieson elastic staining, 200x. (Ryška and Nová, Unpublished results).

No elastic fibers can be detected in diseased liver when necrosis and inflammation are present. The deposition of elastic fibers is a marker of the healing of chronic active hepatitis and accompanies the appearance of large collagen bundles (Bedossa et al., 1990). There is a good correlation between the amount of elastic fibers in the liver and the duration of the illness (Thung and Gerber, 1982). New elastic fibers formed in active fibrosis can be distinguished from the areas of hepatic parenchyma collapse (Scheuer and Maggi, 1980). The deposition of orcein-positive material similar to that in cholestatic liver disease is found in the liver of patients suffering from Indian childhood cirrhosis (Portmann et al., 1978). Bundles of elastic fibers surround central venules in fibrosis accompanying Stage III of nonalcoholic steatohepatitis fibrosis (Nakayama et al., 2008). Elastic fiber deposition in the peripheral portal tracts is a characteristic feature of idiopathic portal hypertension. Sera obtained from patients with this disease induce elastin expression in vascular endothelial cells. Sera obtained from patients with chronic viral hepatitis or cirrhosis have an insignificant effect (Sato et al., 2011). Elastic tissue hyperplasia is also observed in hepatic schistosomal fibrosis (Andrade and Freitas, 1991). Hepatocellular carcinoma is a complication of decompensated cirrhosis. The accumulation of elastic fibers correlates with the development of carcinoma in advanced fibrosis (Yasui et al., 2016). The deposition of elastic fibers in human cirrhotic liver is illustrated in Figures 2A,B.

Elastic fibers formation in the liver can be experimentally reproduced by prolonged treatment of rats with carbon tetrachloride. Elastic stain or antielastin antibody detect elastin in the septa surrounding regenerative nodules (Kanta and Bartos, 1978; Kanta et al., 2002). The ligation of the common bile duct in rats leads to the deposition of elastin in the enlarged portal zone (Lorena et al., 2004; Guyot et al., 2006). Elastin deposition is observed within a few days after bile duct obstruction, first as dots between cells and then as patches next to collagen fibers (Desmoulière et al., 1997). Sepsis induced in mice by polymicrobial polyperitonitis leads to the proliferation of elastic fibers in the portal tracts and in sublobular veins of the liver of the surviving animals (Gonnert et al., 2012). Granulomas formed in the liver of mice injected with Schistosoma mansoni eggs contain concentric orcein-positive elastic fibers (Silva et al., 2000). The content of ECM structural proteins, collagen, elastin, fibronectin and fibrillin are altered in damaged liver. The resulting changes in tissue stiffness may facilitate regeneration or fibrosis (Klaas et al., 2016).

## Microfibrils of elastic fibers

**Figure 3:**
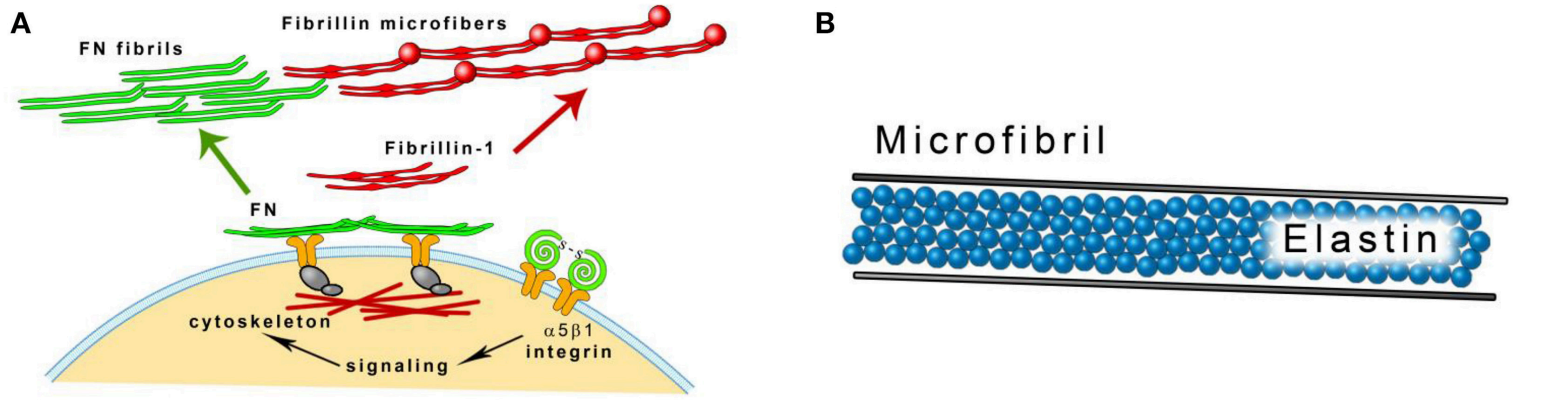
**(A)** Fibrillin microfibrils assembly. Fibrillin-1 is deposited on fibronectin (FN) template that is connected to the cell surface through integrins. Modified from Kinsey et al. (2008). **(B)** A scheme of an elastic fiber consisting of the elastin core and the microfibril mantle.

Microscopically amorphous elastin forms the core of elastic fibers. Microfibrils 10–12 nm in diameter are located on the surface of the fiber parallel to its long axis (Mithieux and Weiss, 2005). Fibrillins are the main protein component of the microfibrils. They are glycoproteins rich in cysteine and their molecular weight is about 350 kDa. The dominating structural element of fibrillins are epidermal growth factor (EGF)-like domains, most of which bind calcium (cb-EGF domains). Calcium cations stabilize the domains and make them resistant to proteolysis. Fibrillin molecules are organized in a head-to-tail alignment (Ramirez and Sakai, 2010). The asembly of fibrillin into microfibrils requires the presence of fibronectin network. Cellular fibronectin is synthesized by fibroblasts and other cells and binds to the cell surface integrins that are connected to the actin cytoskeleton. Binding induces conformational changes in fibronectin molecules. Cellular contractility facilitates the exposing of the domains binding various ECM proteins including fibronectin itself (Mao and Schwarzbauer, 2005; Figure 3A). Isolated microfibrils have a characteristic “beads on a string” appearance. The microfibrils are stabilized by transglutaminase-formed cross-links between interbead regions of the fibrils (Kielty et al., 2002; Wang et al., 2009). Fibrillin-1 binds TE with high affinity and the two proteins may be cross-linked by transglutaminase (Rock et al., 2004). Fibrillin-2 is structurally similar to fibrillin-1 and is also implicated in extracellular tropoelastin deposition (Zhang et al., 1994; Tsuruga et al., 2005). The synthesis of fibrillin-1 parallels the growth of elastic fibers. Fibrillin-2 is produced during fetal life and tissue remodeling. Its expression is high in the fibroblasts isolated from healing wounds (Brinckmann et al., 2010; Ramirez and Sakai, 2010).

Three stages in the development of the elastic fiber can be distinguished by histological methods. Oxytalan fibers are composed of 10–16 nm diameter microfibrils, elaunin contains both microfibrils and amorphous material. In the mature elastic fiber, amorphous material accumulates and forms the central core (Garner and Alexander, 1986; Figure 3B). Oxytalan, elaunin and elastic fibers are all observed in developing alcoholic fibrosis of the liver (Porto et al., 1990b). In normal liver, fibrillin-1 colocalizes with elastin in vessel walls and in portal tract connective tissue. It can also be detected in the absence of elastin. In cirrhotic liver, it can be found in association with elastin in fibrotic septa surrounding regenerative nodules and alone in the perisinudoidal spaces (Dubuisson et al., 2001). Fibrillin-1 is often coexpressed with elastin in children cholestatic diseases but it has a wider distribution in the liver (Lamireau et al., 2002).

## Proteins associated with elastic fibers

**Figure 4:**
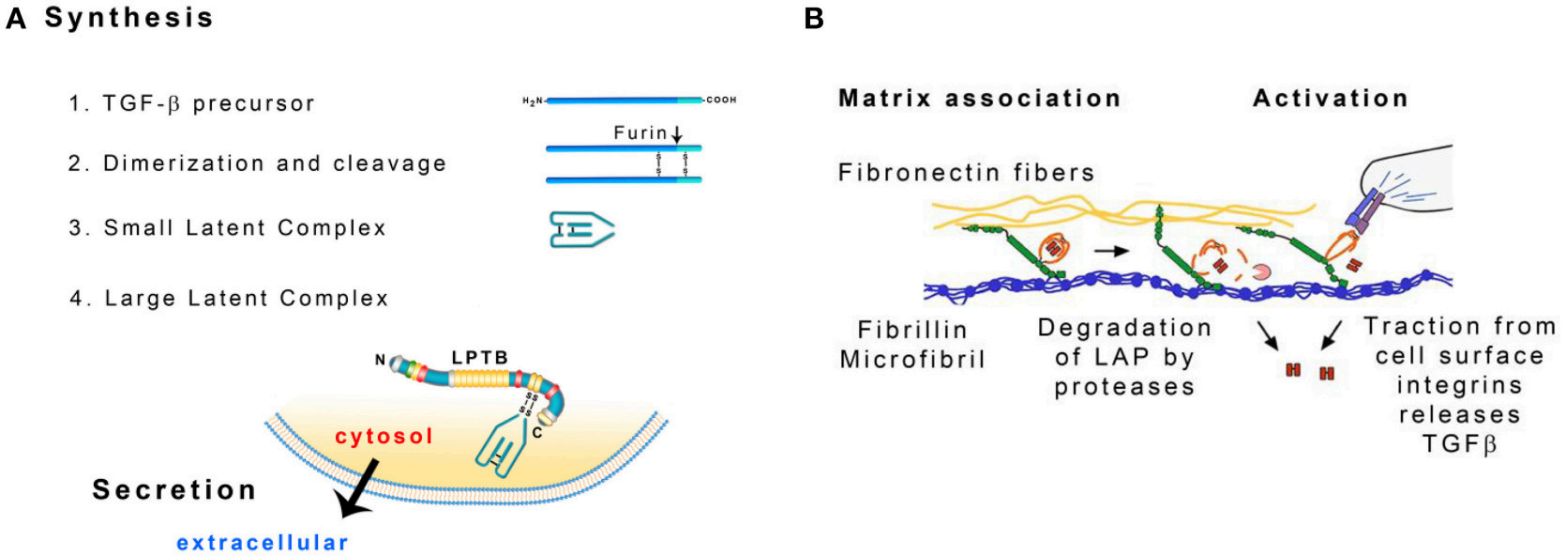
**(A)** Formation of the large latent complex (LLC). TGF-β precursors dimerize and the dimers are cleaved by furin protease. The liberated fragments form the small latent complex (SLC) that contains noncovalently bonded TGF-β. SLC is attached to the latent TGF-β binding protein (LTBP). Modified from Hayashi and Sakai (2012). **(B)** Possible ways to release TGF-β from the Large Latent Complex: degradation of the latency-associated peptide (LAP) by proteases and distortion of the LAP by cellular traction. Modified from Robertson et al. (2015).

A number of proteins are associated with fibrillin microfibrils and influence the properties of elastic fibers (Baldwin et al., 2013). Latent transforming growth factor-β binding proteins (LTBPs) are large glycoproteins (m.w. 125–240 kDa) structurally similar to fibrillin. The family of LTBPs has four members that colocalize with fibrillin microfibrils (Zilberberg et al., 2012; Robertson et al., 2015). LTBPs with the exception of LTBP-2 are secreted from most cells as a large latent complex (LLC) with the latency-associated peptide (LAP)-dimer and the transforming growth factor-β (TGF-β)-dimer (Koli et al., 2001; Figure 4A). LLC attaches to fibrillin during the assembly of microfibrils (Massam-Wu et al., 2010).

LAP and TGF-β are synthesized as a single polypetide chain. Two chains associate to form a dimer that is then cleaved by the endoprotease furin (Koli et al., 2001). As a result LAP dimer (80 kDa) surrounds TGF-β dimer (25 kDa). TGF-β is noncovalently bonded to LAP and LAP is bonded to LTBP through disulfide bonds. LAP prevents TGF-β from interacting with its receptor and signaling. The complex of LAP and TGF-β is called a small latent complex (SLC) (Hayashi and Sakai, 2012; Figure 4A).

Cultured human liver myofibroblasts (MFBs) isolated from liver biopsies express all components forming the large latent complex of TGF-β: four LTBP isoforms and their splice variants, LAP and three TGF-β isoforms, suggesting the importance of TGF-β activity in diseased liver (Mangasser-Stephan et al., 2001). TGF-β1 promotes activation and myofibroblastic differentiation of hepatic stellate cells (HSCs), the key event in liver fibrogenesis (Hayashi and Sakai, 2012). LTBP-1 mRNA can be detected in fibrotic septa in rat liver. It is synthesized by HSCs transdifferentiating into myofibroblasts. TGF-β can be released from the complex with LTBP by plasmin treatment (Breitkopf et al., 2001). As TGF-β is expressed throughout the hepatic parenchyma, LTBP-1 present in the portal spaces may bind TGF-β and target it to these areas (Corchero et al., 2004). LTBP-1 knockout mice are less prone to hepatic fibrogenesis induced by bile duct ligation (Drews et al., 2008).

TGF-β needs to be activated before it exerts its biological activity. The interaction between LAP and TGF-β may be disrupted *in vitro* by extreme pH, chaotropic agents or heat treatment (Koli et al., 2001). LTBP may be cleaved by proteases in cell culture. Plasmin, elastase and a variety of matrix metalloproteases degrade this protein (Dallas et al., 2002). Cellular receptors integrins may serve to bring metalloproteases MT-1 MMP, MMP-9 or MMP-2 into the vicinity of the LLC that is attached to a microfibril. LTBP and LAP are then cleaved and TGFβ is released (Wipff and Hinz, 2008). Traction forces generated by the cells may also facilitate TGF-β release. The αv integrins on the cell surface recognize the arg-gly-asp (RGD) amino acid sequence present in LAP and bind the whole complex. LTBP is anchored in the mechanically resistant matrix and when MFBs contract, the conformation of LAP may change and release TGF-β (Wipff and Hinz, 2008; Figure 4B). According to an alternative mechanism, fibrillin fragments released by proteolysis may inhibit the interaction of fibrillin and LTBP-1 leading to the release of LLC and increase in active TGFβ concentration (Chaudhry et al., 2007).

Thrombospondin-1 may bind to the SLC and the resulting ternary complex of thrombospondin, LAP and TGF-β is biologically active (Murphy-Ullrich and Poczatek, 2000). HSCs are stimulated by platelet derived growth factor (PDGF)-BB to proliferation and migration. The profibrogenic cytokine PDGF-BB stimulates the coexpression of TGF-β1 and thrombospondin-1 in these cells (Breitkopf et al., 2005). In cholestasis, latent TGF-β is activated by thrombospondin-1 whose expression in hepatocytes is induced by bile acids (Myung et al., 2007). LTBP-2 does not form covalent complexes with latent TGF-β but it competes with LTBP-1 for binding sites on fibrillin-1. It is abundant in developing, elastin containing tissues and may negatively modulate LLC storage in the ECM (Hirani et al., 2007).

TGF-β1 is a major profibrogenic cytokine in the liver. It is produced by sinusoidal endothelial cells and Kupffer cells in normal liver. Activated HSCs become the principal source of TGF-β in injured liver and are its primary target. The cytokine stimulates HSCs proliferation, their dedifferentiation to MFBs and ECM synthesis (Bissell et al., 2001; Dooley and ten Dijke, 2012). TGF-β hinders DNA synthesis in hepatocytes and causes their apoptosis or epithelial to mesenchymal transition. It has a bipartite role in hepatocellular carcinoma. It suppresses tumor growth at early stages but it promotes tumor invasiveness and metastasis later (Dooley and ten Dijke, 2012; Meindl-Beinker et al., 2012). TGF-β1 increases fibrillin-1 expression in cultured liver fibroblasts (Lorena et al., 2004).

Fibrillin binds a few members of bone morphogenetic protein (BMP) family (Sengle et al., 2008; Ramirez and Sakai, 2010). Cytokines TGF-β and BMP belong to the TGF-β superfamily. Because of the important role they play in fibrosis they are considered as potential therapeutic targets. The actions of these cytokines are mediated by the intracellular proteins Smads (Munoz-Félix et al., 2015). BMPs regulate cell proliferation, apoptosis, differentiation and migration and have been associated with liver diseases and regeneration (Sugimoto et al., 2007; Herrera et al., 2012).

Microfibril-associated glycoproteins 1 and 2 (MAGP-1, -2) attach to fibrillin. Their molecular weight is small, about 25 kDa. MAGP-1 binds TE and members of TGF-β and BMP family. It may modify TGFβ signaling and tissue homeostasis (Weinbaum et al., 2008; Mecham and Gibson, 2015). Elastin microfibril interface-located proteins (EMILINs) are a family of ECM proteins. EMILIN-1 binds to elastin ad fibulin-5 and is adhesive to cells. It is located at the surface of elastin core. Elastogenesis is impaired in EMILIN-1 deficient mice. Cells lose close contact with elastic fibers, which negatively influences their morphology (Zanetti et al., 2004). EMILIN-1 has not yet been detected in the liver. Microfibrillar-associated proteins (MFAPs) are low molecular weight proteins different from MAGPs (Mecham and Gibson, 2015). MFAP4 is a glycoprotein that binds TE and fibrillin-1 and -2 and the crosslinking amino acid desmosine *in vitro*. It promotes TE coacervation and is necessary for microfibril assembly (Kasamatsu et al., 2011; Pilecki et al., 2016). It is abundant in HSCs and in fibrotic septa of human cirrhotic liver (Mölleken et al., 2009). The expression of MFAP4 in fibrotic liver increases with fibrosis stage both on gene transcript level and on protein level (Bracht et al., 2015).

Fibulins are a family of glycoproteins that comprises proteins with large molecular weight, fibulin-1 (90–100 kDa) and fibulin-2 (200 kDa), and proteins much smaller, fibulin-3, -4, and -5 (50–60 kDa). Their characteristic feature is the presence of calcium-binding epidermal growth factor-like (cbEGF) domains and the fibulin-type C-terminal region. All of them bind TE but they have different roles in elastic fibers formation (Kobayashi et al., 2007). Fibulin-1 is present in the amorphous core of the elastic fiber (Roark et al., 1995), fibulin-2 colocalizes with fibrillin-1 (El-Hallous et al., 2007). The synthesis of fibulin-2 and elastin is highly coordinated (Hunzelmann et al., 2001). Fibulin-4, LOX, and TE form a ternary complex. This complex regulates LOX activation and elastin crosslinking before elastin deposition on the microfibrils. The lack of fibulin-4 causes down-regulation of TE expression in fibroblasts and the formation of irregular elastin aggregates instead of elastic fibers (McLaughlin et al., 2006). The knock-down of fibulin-4 expression in human fibroblasts is accompanied by reduced expression of TE mRNA (Chen et al., 2009). Fibulin-5 is deposited on fibrillin-1 microfibrils in the culture of human fibroblasts (Hirai et al., 2007). It associates with elastin globules and facilitates their deposition on microfibrils (Choudhury et al., 2009). Fibulin-5 gene is induced only in elastin producing cells. Fibulin-5 mRNA expression follows the expression of tropoelastin mRNA and is decreased when the cells are transfected with tropoelastin siRNA (Tsuruga et al., 2004).

Fibulin-1 and -2 are detectable in portal areas of normal rat liver. Their expression increases in acutely injured liver. Fibulin-1 is detectable in hepatocytes, activated HSCs and MFBs but fibulin-2 expression is restricted to MFBs. Fibrotic septa in cirrhotic liver are positive both for fibulin-1 and -2 (Knittel et al., 1999; Piscaglia et al., 2009). Fibulin-2 mediates the action of TGF-β in myocardial fibrosis (Khan et al., 2016) but it is not known if it functions in this way in the liver. The expression of fibulin-5 in liver biopsies of patients with hepatitis B or C associated fibrosis increases with fibrosis stage (Bracht et al., 2015). Fibulin-5 can frequently be detected in the major portal vein branches in patients with idiopathic portal hypertension and its distribution corresponds to that of elastic fibers (Sato et al., 2008).

Lysyl oxidase (LOX) is involved in crosslinking both collagen and elastin. Fibulin-4 interacts with lysyl oxidase (LOX) propetide, tethers LOX to TE and facilitates elastin crosslinking (Horiguchi et al., 2009). Fibulin-5 targets lysyl oxidase-like protein 1 (LOXL1) to the sites of elastogenesis. Binding to fibulin-5 brings LOXL1 into juxtaposition with its TE substrate. LOXL1 is closely associated with elastic fibers in contrast to LOX that is distributed more broadly (Liu et al., 2004). LOX can be detected in fibrotic septa in experimental liver fibrosis (Siegel et al., 1978) and in human viral hepatitis and primary biliary cirrhosis (Vadasz et al., 2005). Members of LOX family are present in HSCs and in portal fibroblasts of healthy and injured liver (Perepelyuk et al., 2013).

Clusterin is an extracellular chaperone that forms complexes with proteins via hydrophobic interactions and stabilizes them on their way to correct folding (Poon et al., 2002). It is present on the outside of abnormal elastic fibers in skin disorders and may protect them from degradation (Aigelsreiter et al., 2013). Clusterin is present in bile in cholestatic diseases. It colocalizes with elastic fibers in liver portal tracts but these complexes are absent from the areas of pericellular fibrosis. Collagen, hepatocytes or cholangiocytes are not stained with clusterin antibodies (Aigelsreiter et al., 2009). It is not clear if clusterin only labels elastic fibers or if it supports their deposition.

Proteoglycans contain a protein core substituted with glycosaminoglycan chains. They are categorized according to their molecular weight and the composition of the glycosaminoglycans. Proteoglycans of various composition, perlecan, versican, and decorin are attached to fibrillin microfibrils (Ramirez and Sakai, 2010). Negatively charged glycosaminoglycan chains interact with positively charged amino groups of lysine residues in TE which may change when the amino groups are oxidized by LOX (Broekelman et al., 2005). Heparan sulfate proteoglycans regulate microfibril assembly and elastin deposition on microfibrils (Cain et al., 2008). Chondroitin sulfate proteoglycans in excess cause abnormal deposition of fibrillin-1 and decreased production of TE and fibulin-5 (Ikeda et al., 2009). Small leucine-rich chondroitin sulfate proteoglycans biglycan and decorin may have a role in the formation of elastic fiber. Biglycan forms a ternary complex with TE and MAGP-1 (Reinboth et al., 2002), decorin forms a complex with fibrillin-1 and MAGP-1 (Trask et al., 2000).

Proteoglycan content in cirrhotic human liver increases significantly. The content of heparan sulfate that prevails in normal liver increases two-fold and the increase in dermatan sulfate and chondroitin sulfate is even more dramatic. Activated HSCs are the principal site of their synthesis (Gressner et al., 1994). Hepatocytes, bile ductular cells and Kupffer cells contribute to proteoglycan synthesis in cholestasis (Roskams et al., 1996). Perlecan and syndecan-1 are localized in the sinusoids of healthy human liver, perlecan is deposited in the fibrotic septa in cirrhotic liver (Tátrai et al., 2010). Perlecan and decorin are also deposited in rat liver and they are expressed in HSCs activated *in vitro* by culture on plastic (Gallai et al., 1996). However, the association of proteoglycans with elastin synthesis and deposition has not been studied in the liver.

## Elastin synthesis and degradation

Elastin is expressed both in portal fibroblasts and in HSCs. Elastin can be detected in isolated portal fibroblasts by immunocytochemistry (Li et al., 2007). Elastin mRNA expression in these cells increases after bile duct ligation (Perepelyuk et al., 2013). The expression of elastin by cell lines derived from portal fibroblasts can be demostrated by mRNA determination, immunoblotting and immunocytochemistry (Fausther et al., 2015). The expression of TE mRNA in HSCs is much lower both in normal and in acutely injured liver (Perepelyuk et al., 2013). TE is expressed in primary HSCs both on mRNA and protein level and is secreted into the culture medium (Kanta et al., 2002). TE can be detected by immunocytochemistry in primary HSCs (Pellicoro et al., 2012). Ethanol stimulates HSC transdifferentiation by causing changes in chromatin structure. The expression of ECM proteins including elastin is increased in this process (Page et al., 2015). Overexpression of miR-29a leads to downregulation of elastin and other ECM proteins in HSC (Li et al., 2011).

Fibrotic septa in the liver are strongly positive for elastin that colocalizes with α-SMA containing myofibroblasts (Kanta et al., 2002; Guyot et al., 2006) but elastin is not detected in activated HSCs in the parenchyma (Lorena et al., 2004). HSCs give rise to 82–96% of myofibroblasts in models of both toxic and cholestatic fibrosis (Mederacke et al., 2013). Portal fibroblasts are the major source of myofibroblasts in early phase of cholestatic liver injury. However, their relative contribution to septa formation decreases as HSCs become activated and attracted chemotactically to the portal spaces (Kinnman and Housset, 2002; Iwaisako et al., 2014; Lua et al., 2016). The majority of elastin present in the fibrotic septa may be a product of HSC-derived MFBs.

Elastin expression is regulated mostly on transcriptional and posttrancriptional level. A number of cytokines participate in elastin regulation in developing and diseased organs. TGF-β1 is one of the most important (Sproul and Argraves, 2013). Mechanical forces play a role in the regulation of elastin synthesis. Collagen and elastin synthesis increases in pulmonary arteries during a period of hypoxic pulmonary hypertension. These changes are reversible but persist if the hypertension becomes chronic (Poiani et al., 1990; Durmowicz et al., 1994). Stretching of aortic smooth muscle cells (SMCs) on culture dishes with deformable bottoms or centrifugation of fibroblasts in cell culture flasks leads to the activation of tropolastin gene and increased tropoelastin synthesis (Sutcliffe and Davidson, 1990; Redlich et al., 2004). Liver stiffness reflects the progression of liver fibrosis. Median area ratios of collagen and elastin fibers correlate well with increasing liver stiffness measured by transient elastography (Abe et al., 2013). Increased stiffness of damaged liver which contributes to HSC activation initially results from a combination of hepatocyte edema, bilirubin elevation and intrahepatic collagen deposition (Dechêne et al., 2010). β-aminopropionitrile, the inhibitor of LOX, partially abolishes the increase in liver stiffness and α-SMA expression (Georges et al., 2007). Elastin fibers are formed later (Klaas et al., 2016). Fibrillin-1 expression in portal fibroblasts and in liver MFBs cultured in restrained collagen gel lattices is larger than that in the cells embedded in freely floating lattices (Lorena et al., 2004).

Elastin is synthesized during fetal life and in the postnatal period. As a result of crosslinking it becomes an insoluble and extremely stable protein. It is the most stable component of ECM. In spite of that it is slowly degraded and small amounts of elastin peptides (micrograms/ml) circulate in the blood sera of healthy individuals (Shapiro et al., 1991; Robert and Labat-Robert, 2014). ECM remodeling is mediated primarily by matrix metalloproteinases (MMPs) containing zinc in their active center. MMP-2 (gelatinase A), -7 (matrilysin), -9 (gelatinase B), and -12 (elastase) are active against elastin. Neutrophil elastase is a serine protease, most cathepsins cleaving elastin belong to cysteine proteases (Qin, 2015). Proteases degrading elastin often cleave fibrillin microfibrils (Sherratt, 2009). Physiological elastin synthesis and degradation in the adulthood occurs in the involuting uterus (Starcher and Percival, 1985). Large amounts of elastin are degraded and elastin synthesis may be reactivated in pathological conditions, e.g., in lung emphysema. Elastin fragments produced by the action of macrophage elastase, MMP-12, attract peripheral blood monocytes (Houghton et al., 2006). Desmosine content in rat cirrhotic liver, which is a measure of mature elastin, increases but elastase-like activity in the organ does not change (Velebný et al., 1983). CCl_4_ and thioacetamide cause severe liver injury both in WT and MMP-12^−∕−^ mice but the accumulation of elastin is much more pronounced in MMP-12 knockout mice. Elastase produced by a subset of hepatic macrophages degrades elastin in the liver and its deficiency leads to enhanced elastin accumulation in experimental liver fibrosis. No spontaneous fibrosis is observed in either group of animals but, after long-term treatment with thioacetamide, increased accumulation of collagen is observed in knockout compared with WT mice (Pellicoro et al., 2012).

## Regulatory role of elastin

Elastic fibers provide elasticity and resilience to the organs that are subject to repetitive distension and physiological stress (aorta, lungs, skin). However, this is not their only function. The regulatory role of TE, elastin polymer and products of elastin degradation have become prominent in recent years (Lannoy et al., 2014). Elastin acts as an autocrine factor that inhibits the proliferation of vascular SMCs in arteries and keeps them in a quiescent contractile state. Mice lacking elastin gene (Eln^−/−^) die from occlusive fibrocellular pathology caused by subendothelial proliferation and accumulation of vascular SMCs in early neonatal life. SMCs isolated from newborn Eln^−/−^ pups proliferate at a much higher rate than Eln^+/+^ cells. Eln^−/−^ SMCs show a remarkable paucity of actin stress fibers that can be reversed by recombinant TE treatment. TE inhibits SMC migration and proliferation in culture (Karnik et al., 2003a). Insoluble elastin incorporated into collagen scaffolds reduces their stiffness in a concentration dependent manner and modulates SMC phenotype toward a contractile state (Ryan and O'Brien, 2015; Nguyen et al., 2016). Eln^+/+^ cells have a spindle-shaped morphology with prominent stress fibers in contrast to the epitheloid-like morphology of Eln^−/−^ cells (Karnik et al., 2003b).

Hydrophilic domains of tropoelastin rich in lysine are often involved in cross-link formation and the products of elastolytic cleavage are fragments of the hydrophobic parts of the molecule containing the hexapeptide val-gly-val-ala-pro-gly (VGVAPG) and other peptides containing GXXPG sequence where X is a hydrophobic amino acid. These peptides are biologically active (Almine et al., 2010). Elastin peptides (EP) sometimes called elastokines may bind to the elastin receptor complex consisting of EBP, neuraminidase and protective protein-cathepsin A and located on the cell surface (Adair-Kirk and Senior, 2008; Maurice et al., 2013). Integrin αvβ3 binds the sequence RKRK at the C-terminal of TE (Rodgers and Weiss, 2004; Almine et al., 2013). Galectin-3 occurring on breast carcinoma cells can be regarded as the third elastin binding receptor (Ochieng et al., 1999). EP undergo phase transition and self-assembly above a certain critical temperature which increases EP concentration and makes their action more effective (Yuan and Koria, 2016).

Fragments of ECM including elastin are chemotactic for monocytes and polymorphonuclear leucocytes (Hauck et al., 1995; Houghton et al., 2006). They induce changes in gene expression of inflammatory cells (Adair-Kirk and Senior, 2008). EP stimulate MMP-12 secretion by fibroblasts. This proteinase may in turn produce more elastokines (Almine et al., 2013). The expression of proinflammatory cytokines TNF-α, IL-1β, and IL-6 in monocytes is suppressed by EP and elastin fragments thus modify inflammation (Baranek et al., 2007).

Elastin hydrolysate and EP stimulate the proliferation of fibroblasts and SMC (Chatron-Colliet et al., 2015; Shiratsuchi et al., 2016) and enhance angiogenesis (Robinet et al., 2005). EP stimulate production of some MMPs (Robinet et al., 2005; Duca et al., 2007) and decrease elastin synthesis (Wachi et al., 1995). Protein kinases ERK1/2 are involved in MMP regulation (Duca et al., 2007). TE modulates the release of vascular endothelial growth factor (VEGF) and connective tissue growth factor (CTGF) from SMC stimulated by TGF-β1 (Reddel et al., 2013).

## Elastic fibers in the diagnosis of liver diseases

Elastin that accumulates in the liver with advancing fibrosis can be used to diagnose the disease. Magnetic resonance with an elastin-specific contrast agent containing gadolinium may be used as a noninvasive diagnostic technique (Ehling et al., 2013). Accumulation of elastic fibers in the liver is a major predictor for the development of hepatocellular carcinoma, independently of collagen fibers (Yasui et al., 2016). MFAP4 expression is low in the liver compared to elastic tissues (Wulf-Johansson et al., 2013). However, MAFP4 concentration in the plasma of patients with chronic hepatitis and cirrhosis is increased and positively correlates with liver stiffness measured by transient elastography. The increase in plasma MFAP4 level in patients with liver disease is higher than that observed in patients with heart or other diseases (Sækmose et al., 2013, 2015).

ECM markers, degraded collagen III, collagen V and elastin, correlate with galactose elimination capacity and with the degree of portal hypertension (Leeming et al., 2013). The concentration of circulating EP may reflect ECM remodeling and predict survival time in patients with transjugular intrahepatic porto-systemic shunt (Nielsen et al., 2015). Clusterin is present in serum. Its concentration decreases in patients with alcoholic cirrhosis and is correlated with their survival (Høgåsen et al., 1996). The presence of desmosines, degradation products of elastin, in urine is a potential diagnostic marker of liver fibrosis (Afdhal et al., 1997; Stone, 2000).

## Conclusions

The formation of fibrotic septa is regarded as a negative outcome of liver diseases. Elastic fibers contribute to the stability of the septa. All aspects of the presence of elastic fibers in the liver are not known, however. The synthesis of individual components of elastic fibers in the liver and their assembly is likely to follow the general pattern of elastic fibers formation in the organism. The knowledge of the regulation of elastogenesis obtained in the studies done in other organs may provide a useful inspiration for the study of elastogenesis in the liver. The emerging regulatory role of fibulins is an example. The research done in human cells should be given a priority because of the differences in the splicing of the primary TE gene transcript in various animal species. Elastin and other components of elastic fibers may also be useful as diagnostic tools in hepatology.

## Author contributions

The author confirms being the sole contributor of this work and approved it for publication.

### Conflict of interest statement

The author declares that the research was conducted in the absence of any commercial or financial relationships that could be construed as a potential conflict of interest. The reviewer PK and handling Editor declared their shared affiliation, and the handling Editor states that the process nevertheless met the standards of a fair and objective review.
